# Accelerated Wound Closure - Differently Organized Nanofibers Affect Cell Migration and Hence the Closure of Artificial Wounds in a Cell Based *In Vitro* Model

**DOI:** 10.1371/journal.pone.0169419

**Published:** 2017-01-06

**Authors:** Maximilian Ottosson, Albin Jakobsson, Fredrik Johansson

**Affiliations:** Dept. Biology, Unit of Functional Zoology, Bio-interface Group, Lund University, Lund, Sweden; Universite de Technologie de Compiegne, FRANCE

## Abstract

Nanofiber meshes holds great promise in wound healing applications by mimicking the topography of extracellular matrix, hence providing guidance for crucial cells involved in the regenerative processes. Here we explored the influence of nanofiber alignment on fibroblast behavior in a novel *in vitro* wound model. The model included electrospun poly-ε-caprolactone scaffolds with different nanofiber orientation. Fibroblasts were cultured to confluency for 24h before custom-made inserts were removed, creating cell-free zones serving as artificial wounds. Cell migration into these wounds was evaluated at 0-, 48- and 96h. Cell morphological analysis was performed using nuclei- and cytoskeleton stainings. Cell viability was assessed using a biochemical assay. This study demonstrates a novel *in vitro* wound assay, for exploring of the impact of nanofibers on wound healing. Additionally we show that it’s possible to affect the process of wound closure in a spatial manner using nanotopographies, resulting in faster closure on aligned fiber substrates.

## Introduction

Increased understanding and advances in molecular- and cellular biology in recent years have greatly improved our understanding of the wound healing process [1]. This in turn has helped researchers and clinicians in their development of new improved methods for wound care, along with treatments to enhance the healing of wounds [2, 3]. Also, advances have been made in tissue engineering, and its applications within this field [4, 5].

The healing/regeneration of the skin incorporates a re-establishment of the dermis structure, *i*.*e*. vascularized granulation tissue, before a proper closure of the epidermis (epithelial cells) is meaningful. A limiting factor for the formation of the granulated tissue is the activation of fibroblasts [6]. Triggering fibroblast migration into the wound area may speed up the remodeling of the dermis, and hence pave the way for faster closure of the epithelial layer. In order to accelerate these events, an artificial topographical guidance cue, resembling the native extracellular matrix (ECM) structure can be used.

The use of proper scaffolds is a crucial step in all tissue-engineering applications as it is generally accepted that cells are affected by their local physical environment. In the last decade, electrospun fibers have gained a lot of interest within the field of regenerative medicine, mainly due to the physical resemblance of native ECM fibers [7, 8]. The technique enables the use of many different polymers, both of artificial and natural origin [9]. Furthermore, functionalization of the fiber structure can be tuned to mimic the local cellular environment regarding structure and biochemical recognition sites, e.g. Arg-Gly-Asp (RGD) sequences or whole proteins such as laminin or fibronectin [10].

Additionally, studies have shown that anisotropic orientation of nanofibers have an effect on cell adhesion mechanisms, and guide the process as well as affect the morphology of cells [11, 12]. Of interest for wound healing, it has been demonstrated that fibroblasts change the expression of matrix components e.g. elastin, collagen and fibronectin depending on the substrate morphology [11].

In this study, we have focused on exploring the pure topographical effects that an underlying scaffold material may have on fibroblasts *in vitro*. A custom made, stencil based wound assay with L929 murine fibroblasts was used to study the closure of circular wounds over time. There are two main mechanisms for closing a wound/hole of a cell layer: Cell migration and the purse-string contraction of a pluricellular actomyosin cable [13]. The latter is believed to rely partly on myofibroblasts and be the main mechanism for small gaps while the former is the dominating mechanism for macroscopic wounds, and the focus of this study [14].

Traditionally cell migration experiments are performed using so called “scratch” assays, in which a wound is induced into a monolayer of cells by scratching, using for example a pipette tip. As the scratch is made manually it is inherently difficult to reproduce the model in an accurate manner. The process of scratching also physically damages any underlying surface, which makes it unsuitable for our experiments. There are approaches to circumvent the issues coupled to the scratch assays, such as the Oris^™^ Cell Migration and Invasion Assays (Amsbio, UK), or the Radius^™^ Cell Migration Assay (Cell Biolabs Inc, USA). These assays utilize cell stoppers or gels to create cell-free-zones into which migration can occur upon removal of the gel/stopper. Gel component residues might affect the underlying scaffold properties and therefore we used a stopper based assay for the present study.

To make sure the assay would be compatible with our nanofiber scaffolds we designed and 3D-printed our own inserts with a removable cell-stopper in biocompatible polylactic acid (PLA). Nanofiber scaffolds produced through electrospinning were then mounted on the inserts and prepared for cell seeding.

Poly-ε-caprolactone (PCL) was chosen as the scaffold material since it is a well-described, biodegradable, non-toxic polymer [15]. Additionally, PCL has been approved for medical applications related to wound care (*i*.*e*. Capronor^®^, SynBiosys^®^, Monocryl^®^ sutures), by the Food and Drug Administration since the 1980’s. Furthermore, by using an artificial polymer, all scaffolds will have identical surface chemistry throughout all batches, favoring the study of topographical effects alone.

Following seeding of fibroblasts we evaluated cell mobility, viability and morphology using routine nuclei- and cytoskeleton staining and a biochemical cell viability assay. Based on previous studies of cell behavior on oriented nanofibers [16–20] we hypothesized that an oriented scaffold would increase directed cell mobility as well as favor cell polarization and elongated cell morphologies due to directed contact guidance, compared to random fiber scaffolds and flat control surfaces, and thus accelerate the wound closure process.

## Materials and Methods

### Materials

PCL (M_n_ = 80 000 Da, Sigma-Aldrich, USA), Acetone (99.6%, VWR International AB, Sweden), EtOH (99.6%, CCS Healthcare AB, Sweden), RPMI-1640 (Gibco^®^, Thermo Fisher Scientific, USA), Penicillin-Streptomycin (Merck Millipore, Germany), L-glutamine (Merck Millipore, Germany), Paraformaldehyde (PFA, Sigma Aldrich, USA), Triton X-100 (Sigma, USA). bisBenzimide H33258 (Sigma, USA). Phalloidin Alexa Fluor 633 (Invitrogen, Molecular probes, USA). AlamarBlue Cell Viability Assay (Thermo Scientific, USA). Any other chemicals used were of analytical grade.

### Nanofiber scaffold preparation

PCL was dissolved in acetone at a concentration of 15% (w/v) on a hot plate (Heidolph MR 3001K) at 50°C over the course of two hours, and under constant magnetic stirring. For electrospinning of randomized nanofibers the solution was loaded in a 1 mL plastic syringe equipped with a 22 G, blunt tip metallic needle (Nordson EFD). The solution was then spun to a 10 x 15 cm aluminum collector covered with aluminum foil using a HCP 35–35000 DC power supply (FUG) with an electric potential set to 17.5 kV and an Aladdin-1000 laboratory syringe pump (World Precision Instruments) set to a constant flow rate of 2 mL/h. The working distance was set to 25 cm and the spinning time to 15 min. Aligned nanofibers were spun using the same setup but with aluminum foil mounted on a rotating aluminum cylinder (Ø = 15 cm) set to 1500 RPM, an electric potential of 18 kV, a flow rate of 3 mL/h, a working distance of 20 cm and a spinning time of 20 min. A summary of the process parameters is presented in Table 1. The spinning process is reproducible and scaffolds were produced over the course of several spinnings on different days. All spinnings were performed at room temperature (RT) and at a relative air humidity of 30–35%. The electrospun scaffolds were finally placed in a desiccator for >24h prior to cell culturing to ensure removal of all remaining solvents.

**Table 1:** The electrospinning process parameters used to produce random- and aligned nanofiber scaffolds.

Sample	PCL concentration (%, w/v)	Working distance (cm)	Applied voltage (kV)	Polymer flow rate (ml/h)	Rotational speed (RPM)
Random	15	25	17.5	2	-
Aligned	15	20	17.5	3	1500

### Nanofiber scaffold characterization—morphology, distribution and alignment

To evaluate detailed fiber morphologies, a SU3500 scanning electron microscope (SEM) (Hitachi, Japan) was used at an accelerating voltage of 10 kV. Scaffold samples were coated with a 15 nm layer of Au/Pd using a 108 auto sputter coater (Cressington Scientific Instruments Ltd., UK). Images were taken at 800 and 1000 times magnification, from which the diameter and angle of 600 fibers per sample type (random or aligned fibers) were measured manually using ImageJ software (NIH, USA). Additionally 60 pores per sample type were measured (n = 3 with 20 replicates per group) to calculate the average pore area.

Scaffold thickness was measured (n = 6) using an Olympus BX40 microscope (Olympus, Japan) with a thickness measurement tool (Heidenhain, Germany) and porosity (ε) was calculated using measured average sample density and standard density of the scaffold material (ρ_0_ = 1.145 g/cm^3^) as shown in Eq 1.

$$\varepsilon =\left(1-\;\frac{\rho }{\rho_{0}}\right)\;\cdot \;100$$ (1)

where ρ is the average sample density and ρ_0_ is the standard density.

### Hydrophobic properties

Static water contact angle measurements were performed to investigate any difference in hydrophobic properties between the different scaffolds. A 10 μL drop of ultrapure deionized H_2_O was placed on the substrate surface and the contact angle measured using a Canon EOS 600D digital camera, mounted on a custom made goniometer consisting of a microscope and a moveable stage. The process was repeated at five points per sample and the images taken were analyzed using ImageJ software with the DropSnake plugin [21].

### Manufacturing custom inserts

To observe cell mobility a novel *in vitro* wound model was set up using CAD and 3D printing. The design was made using the free CAD software OpenSCAD and the printing was done using a Makerbot Replicator 2 (Makerbot Industries, USA). In this way, custom PLA inserts were manufactured onto which the nanofiber scaffolds were mounted. The inserts were designed with a removable part made up of four pillars (Ø = 1.5 mm) in contact with the fiber scaffold, working as “cell blockers” which effectively masked the fiber surface and thus created four circular and cell free “wound areas” per scaffold sample. The inserts were designed in two sizes, one to fit in standard 35 mm cell culturing petri dishes (Nunc, Thermo Scientific, USA) for cell mobility experiments and one to fit in the individual wells of standard 24 well plates (Nunc, Thermo Scientific, USA) for cell viability experiments. Scaffolds mounted on these inserts were sterilized using 70% EtOH before being UV treated overnight and subsequently placed in the corresponding dishes. The scaffolds were finally pre-incubated with culturing media for 30 min before cell seeding.

### Cell culture and seeding

The L929 cells were cultured in in the same media throughout the study, which was RPMI1640 medium with 10% fetal calf serum (FCS) and Penicillin-Streptomycin (100 U / mL) at 37°C in a humidified incubator with 5% CO_2_. At the day of seeding the cells were enzymatically detached (trypsin, 100 μl/ml medium) and made into a single cell suspension. The cell suspension was made in the culturing media. Cells were seeded on the three different surfaces, flat (standard 24-well cell culture plate wells or 35 mm petri dishes depending on the experiment), random fiber- and aligned fiber substrates. Cell concentrations and survival timed used for the specific parameter analyzed are given below.

### Cytoskeleton and nuclei staining—morphological analysis

At the end of the experiments, for all stainings, cells were fixed using 4% paraformaldehyde (PFA) in PBS (pH 7.4) for 20 min, rinsed in phosphate buffered saline (PBS), and subsequently permeabilized using 0.25% Triton X-100 in PBS (pH 7.4) for 10 min. Cell nuclei were then stained using bisBenzimide (1:1000 in PBS) for 5 min and actin filaments were stained using phalloidin Alexa Fluor^®^ 633 (1:50 in PBS) for 20 min. All stainings were performed at RT and under dark conditions.

Morphometric cell nuclei analysis was performed on cells (1 × 10^6^ cells/35 mm petri dish) grown for 96 h on the three different surfaces (three independent seeding session, n = 3 replicates/session/group. Images were taken using either an Axio Imager M2 fluorescence microscope (Carl Zeiss, Germany) or an AX70 fluorescence microscope (Olympus Corporation, Japan), and detailed quantitative morphometric analysis of nuclei was performed using ImageJ software (NIH, USA).

### Cell mobility assay

Cells (1 × 10^6^ cells/dish) were seeded on custom inserts placed in 35 mm petri dishes and incubated for 24 h to create a confluent monolayer. After 24 hours the removable part of the inserts was removed, leaving four circular cell-free zones per dish, which were evaluated after 0-, 48- and 96 h using the fluorescent stainings as described above. Micrographs were analyzed using ImageJ software and the MRI wound healing tool plugin [22]. These experiments were repeated six times (n = 6, with 4 replicates per group and time point).

### Cell viability assay and analysis

Seedings were made on the three different surfaces with 5 × 10^4^ cells/well seeded onto scaffolds mounted in the custom made 24 well plate inserts, and placed in the wells of a 24 well plate (Nunc, Thermo Scientific, USA). Cells were analyzed after 48- and 96 h using the alamarBlue Cell Viability Assay according to the manufacturer’s procedure and a Spectramax M2 microplate reader (Molecular Devices, USA). This experiment was repeated twice and with 8 replicates per surface type and time point.

### Statistical analysis

All statistical analysis was performed using GraphPad Prism Version 6 (GraphPad Software, USA). For the multiple group comparisons, a one-way ANOVA with Tukey′s multiple comparison test with a single pooled variance was applied. Null hypothesis was rejected at p<0.05. Data significance is indicated with (*) for p<0.05, (**) p<0.01, (***) p< 0.001 and (****) p<0.0001. Results are reported as mean ± SD (standard deviation).

## Results

### Characterization of fibrous scaffolds

**Fig 1:**
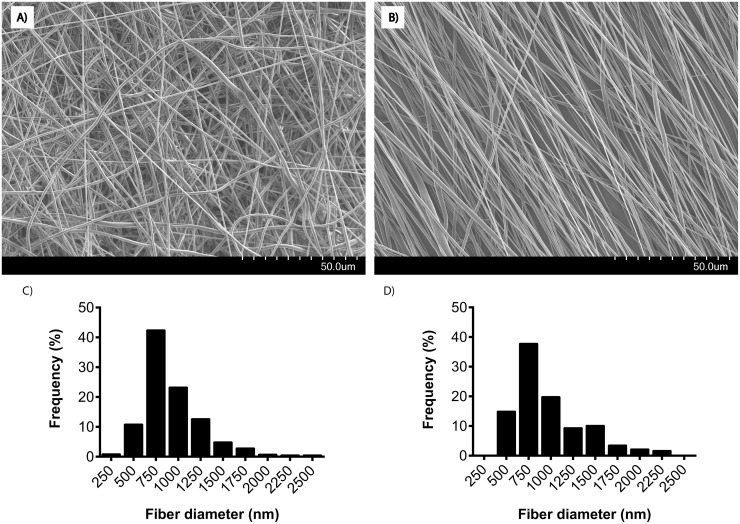
**SEM images of A) random- and B) aligned PCL nanofiber scaffolds**. The histograms display a typical skewed right distribution of fiber diameters for both C) random- and D) aligned scaffolds. The electrospun PCL fibers had similar distribution with median diameters around 750 nm. The random and aligned fiber structures show distinct differences regarding fiber morphology.

**Fig 2:**
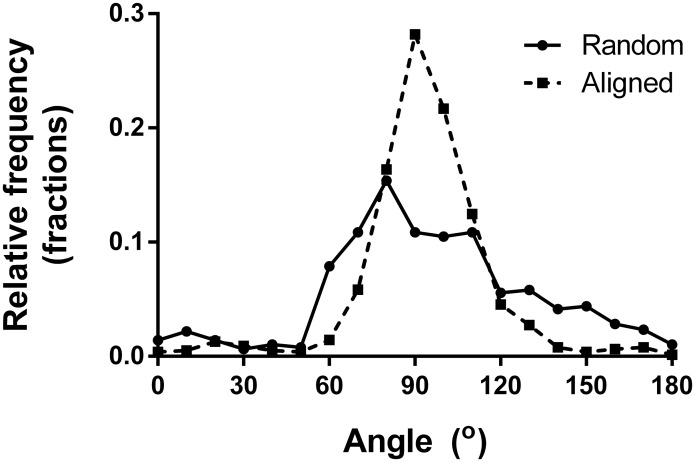
The alignment of individual fibers in each scaffold. The alignment was calculated from 600 fibers per scaffold type and shows a clear difference in overall orientation.

PCL nanofiber scaffolds were produced through traditional electrospinning. Scaffolds are constituted by fibers of either random or aligned orientation. Analysis from SEM images verifies that fibers of similar diameters and distributions were achieved (Fig 1), while the overall orientation clearly differs between the two scaffold types. The resulting scaffold dimensions and properties are presented in (Table 2). To quantify the different alignment of the two scaffolds the angles of 600 fibers per scaffold type were measured manually (Fig 2).

**Table 2:** Results from characterization of both nanofiber scaffold types.

Sample	Fiber diameter (nm)	Sample thickness (μm)	Pore size (μm^2^)	Pore width (μm)	Porosity (%)
Random	935 ± 350	86 ± 25	281 ± 187	13 ± 5	>90
Aligned	986 ± 436	110 ± 18	60 ± 50	3 ± 1	>90

For the characterization, n = 600 fibers/scaffold type were included for fiber diameter analysis, n = 60 pores/scaffold type for pore measurements and n = 3 scaffolds of each scaffold type were included for sample thickness and porosity measurement.

### Surface hydrophobic properties

**Fig 3:**
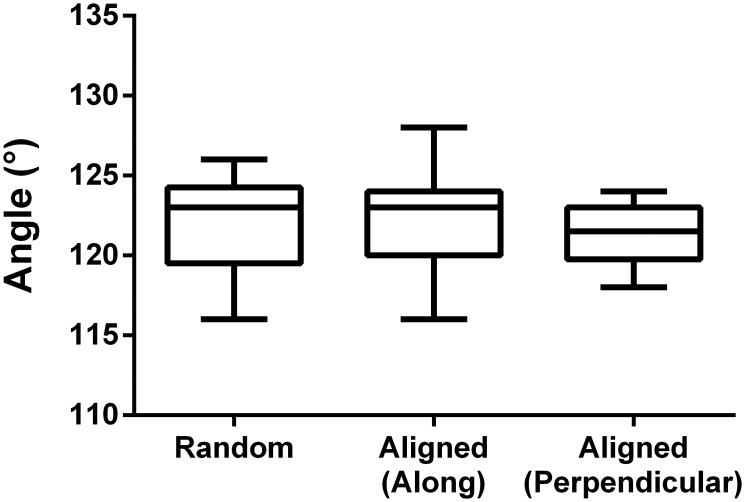
Resulting static water contact angles for the two types of PCL scaffolds. The angle was measured both parallel- and perpendicular to the aligned fibers to exclude any possible discrepancies.

The scaffolds hydrophobic properties were evaluated using static water contact angle analysis. PCL is known to be hydrophobic which our measurements confirmed (Fig 3). A contact angle between 116° and 128° was found, regardless of the fiber substrate fiber orientation, which sometimes can have a marked topographical effect on the drop shape. To enhance hydrophilicity and cytocompatibility prior to cell culture, all samples were pre-soaked in cell culture medium with 10% fetal calf serum for 30 min. To verify the effect of the pre-culture treatment, contact angle measurements were repeated on samples incubated for 30 min in media and consequently air dried. The surface properties changed significantly to highly hydrophilic by this treatment which was further quantified by the contact angles that were too small to be measured, *i*.*e*. close to zero.

### Manufacturing custom inserts

**Fig 4:**
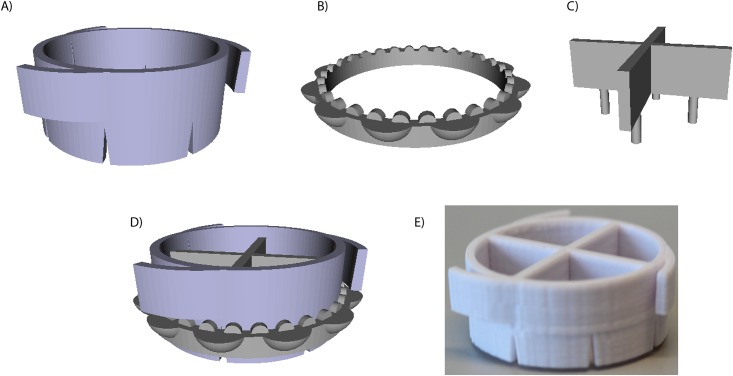
The design of the different parts comprising the custom cell stopper inserts. A) The main part of the insert onto which the scaffold is mounted. B) The toothed ring holding the scaffold in place. C) A removable part with four 1.5 mm pillars acting as cell stoppers. D) All parts assembled into the final insert design. E) The final device as printed in PLA.

Insert design was divided into three parts: the main part of the insert is constituted by a winged ring, designed to flex and secure into place in the vial of choice (either a standard 35 mm dish or the well of a 24 well plate) (Fig 4A). A toothed ring that keeps the fiber scaffold in place on the main part of the insert (Fig 4B) while the removable part, with the cell stopper pillars, fit inside the main part (Fig 4C). The full insert design is depicted in Fig 4D along with the 3D printed physical version (Fig 4E).

The insert preparation, with a total production time of about 15 min per insert, and mounting the fiber scaffolds was easy and reproducible. Insert performance was excellent and “wound areas” created upon removal of cell stopper were similar in size throughout all experiments, even though the underlying structure varied (random/aligned/flat).

### Cell morphology

**Fig 5:**
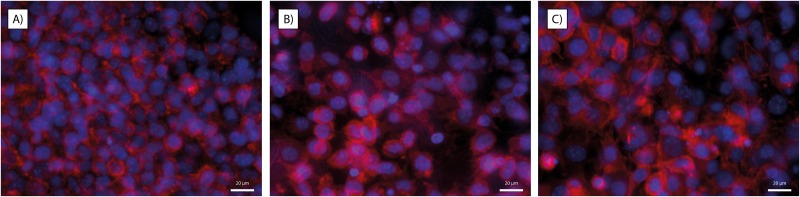
Stainings for nuclei (blue) and actin filaments (red). A) flat- B) random fiber- C) aligned fiber surfaces. Cells have formed confluent monolayers on all surfaces, and cellular morphologies are thus difficult to analyze. Scale bar = 20 μm.

**Fig 6:**
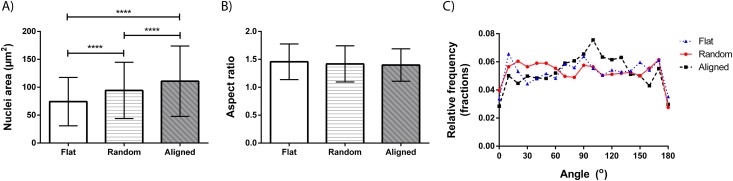
Nuclei analyzes. A) Cell nuclei size differs significantly between cells cultured on different substrates. B) The aspect ratio of the nuclei is however unaffected by the surface and displays a slight elongation on all topographies. C) The orientation of the nuclei long axes shows a clear trend to orient along the aligned nanofibers, which is not displayed on the other topographies.

Due to the high cell seeding density and rapid proliferation, it was difficult to analyze individual cell body morphologies, as they were densely packed in a confluent layer on all surfaces (Fig 5), why cell body morphology was not possible to perform. Instead, cell nuclei were analyzed for orientation, aspect ratio (major axis/ minor axis) and area. Cell nuclei size differs significantly (p < 0.0001) between the different substrates, ranging from smallest on the flat surface (74 ± 43 μm^2^) to the random fiber scaffold (94 ± 50 μm^2^) and finally largest on the aligned fiber scaffold (111 ± 63 μm^2^) (Fig 6A). Aspect ratio measurements showed that all nuclei are slightly elliptical (aspect ratio >1) regardless of underlying surface structure (Fig 6B). However, the alignment analysis based on the nuclei orientation was found to be surface structure dependent. Cells on aligned nanofiber meshes showed higher degree of alignment (along the fiber direction) than on the random fiber or flat surfaces (Fig 6C).

### Cell mobility

**Fig 7:**
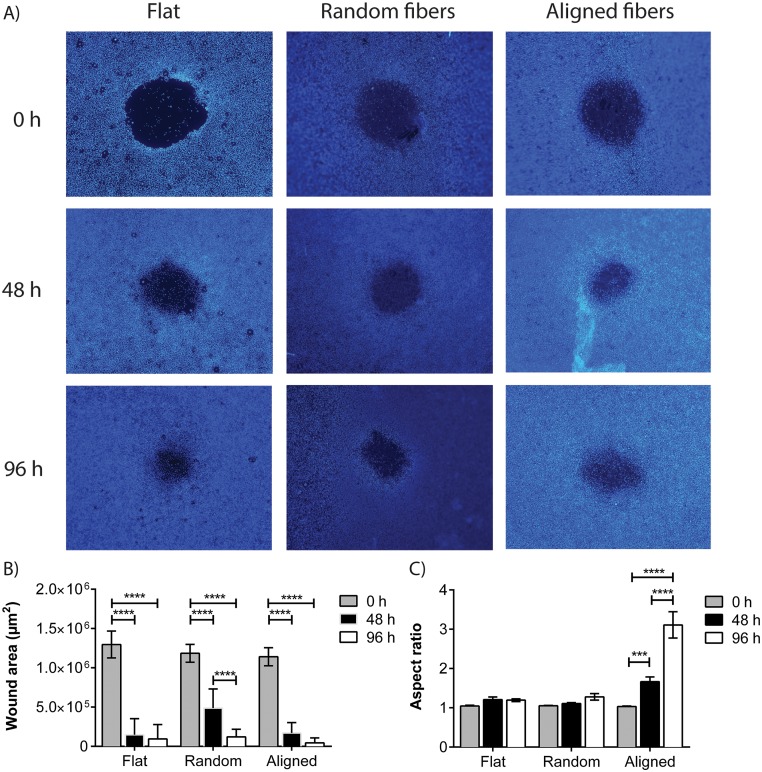
Closing of artificial wounds. A) To visualize the wound closing process, nuclei stainings (bisBenzimide) were used. B) The cell free area of each wound was measured at the different time points. C) Additionally, the aspect ratio *i*.*e*. shape of the wound areas was calculated for each time point and wounds on aligned nanofiber scaffolds appeared to adopt an increasingly elliptical shape over time.

For the purpose of this study, we define mobility as a combination of proliferation and migration, since both occur during the natural wound healing process. To assess the rate of mobility and consequently the wound healing rate, the area of the artificial wound was measured at three different time points. The observed average rate was slowest on the scaffolds comprised of random nanofibers. The wound model closed slightly faster on flat surfaces while the rate of wound closure was found to be fastest on aligned fiber surfaces (Fig 7A and 7B). In addition to the faster pace at which the wound field closed, the spatial manner in which it does so also depends on the surface topography (Fig 7C). While the wounds on the flat control surfaces and random fiber scaffolds maintained their circular shape over time of wound closure, the wounds on the aligned fiber scaffolds closed in a more anisotropic, elliptical fashion.

### Cell viability

**Fig 8:**
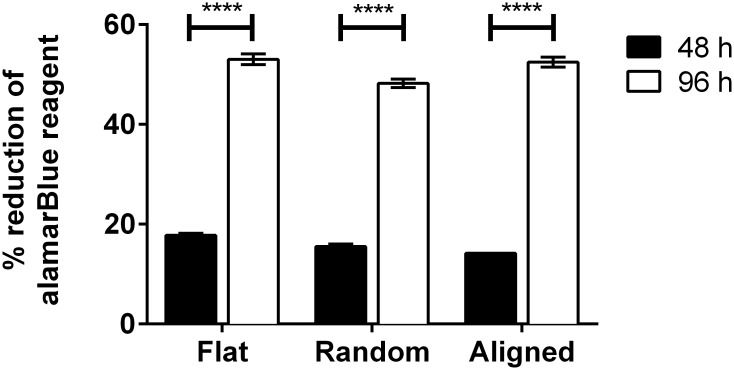
Cell respiratory activity. The percentage of reduced alamarBlue reagent has visibly increased between 48- and 96 h on all topographies. This is to be expected as cells are given serum to keep proliferating throughout the experiment duration. The extent to which they reduce the reagent differs minimally at both time points.

Viability was evaluated using a respiratory based assay, thus indirectly proliferation was measured since the chemical reduction of the reagent is: the activity per cell times the number of cells. A significant increase in reduction can be observed from 47- to 96 h on all three types of topographies, and only slight differences were observed between the three at both time points (Fig 8).

## Discussion

The experimental results gathered throughout this project supports the hypothesis that an oriented scaffold would increase directed cell mobility as well as favor cell polarization and elongated cell morphologies compared to random fiber scaffolds and flat control surfaces, and thus accelerate the wound closure process. This is an important effect for several medical applications where orientation/polarization is of essence.

Today there are numerous types of ECM-mimicking scaffolds used within tissue engineering, such as hydrogels and sponge shaped structures. However, such scaffolds are usually isotropic and provide no guidance cues for cells. In this study we focused on PCL fiber scaffolds produced through electrospinning, since these have been thoroughly evaluated for cell culturing and tissue engineering purposes [23]. PCL has a low degradation rate (many months *in vivo* and even longer *in vitro*)[24] and high elongation parameters, both necessary properties for clinical grafts and implants, where the material is required to be stable over sufficient time, but still enables bending, contraction and expansion [25]. Hence, electrospun PCL scaffolds provides a proper physically and chemically stable platform, suitable for cell culture experiments [26]. However, the material properties (degradation time and functionalization) can be tuned since PCL can be easily mixed and co-electrospun with e.g. natural polymers, such as e.g. collagen, gelatin or chitosan.

There have been several studies on the effect of fiber diameter on fibroblast morphology, mobility, viability and orientation [12, 27]. According to Liu et al. fibers with a diameter less than 0.97 μm does not affect fibroblast orientation. Additionally, Pelipenko et al concluded that fibers in the range of 300–700 nm are most suitable for tissue regeneration since they stimulate fibroblast proliferation, while thinner fibers were shown to affect cell mobility and thus indirectly wound closure in a beneficial manner. Here, we tuned fibers towards average diameters close to the one reported by Liu, *i*.*e*. sub-micrometer, to achieve fibroblast orientation while still maintaining a range of thinner fiber diameters, mimicking natural ECM and supporting the reported cell mobility [12]. The resulting fiber diameters were around 950 nm and highly reproducible regarding both fiber diameter and alignment. However, the intrinsically stochastic mechanism of electrospinning will result in some non-aligned fibers even in the anisotropic set up, which was also found in our studies. Apart from the apparent differences in orientation between the two scaffold types, they primarily differ in pore size (281 ± 87 and 60 ± 50 μm^2^ on random and aligned scaffolds respectively). Both pore sizes should in theory allow cells to migrate into the scaffold, however, pores in the oriented fiber network tend to be very narrow (3 ± 1 μm) which will most likely inhibit any infiltration.

The results from characterizing the scaffolds hydrophobic properties show that PCL nanofiber scaffolds are indeed hydrophobic, regardless of scaffold alignment. As we opt to use the scaffolds for cell culturing, and cells prefer a more hydrophilic environment[28], the hydrophobic properties of PCL should be neutralized. We successfully made the scaffolds hydrophilic and cell supporting by pre-incubating them in cell culture media for 30 min, prior to cell seeding. The effect of the pre-incubation was furthermore verified by contact angle measurements which showed contact angles too small to be measured *i*.*e*. close to zero.

Regarding the production of the mobility inserts, the manufacturing techniques used to create the custom inserts offer a great degree of flexibility. This allowed us to easily modify and iterate through different versions of the insert until a final design was chosen. Using 3D printing we could then reproduce and effectively manufacture as many inserts as needed in a time- and cost effective manner.

For this study, fibroblasts were chosen as they are naturally occupying the ECM structures, producing and remodeling the 3D network of structural proteins such as collagen and elastin and hydrophilic molecules such as glycosaminoglycans (GAGs). This is fundamentally important during injury, where many different cell types, including fibroblasts move towards the site of injury to participate in the wound healing events. Here in this initial study, we used the murine L929 fibroblast cell line, a well characterized cell line in our and others laboratories. The cell line proved to be excellent for this application, but could obviously be exchanged for other cells of interest such as macrophages and keratinocytes.

Concerning the use of the cell nuclei for morphological analysis of the cells, the nucleus size change during the cell cycle, but since the cells are not synchronized the average nucleic size can still be a useful measurement, reflecting the forces acting on the cell. Cell nuclei size on flat surfaces was in accordance with previous observations of fibroblasts, *i*.*e*. around 75 μm^2^ [29]. The cells cultured on fiber scaffolds in general and on aligned fibers in particular seem to have larger nuclei than those on flat surfaces. Nuclei size has previously been demonstrated to depend on the surface topography, and more specifically on the pitch between linear structures. McKee et al. showed for epithelial cells that the largest nuclei were found on a linear topography with a pitch of around 1200 nm (400–2000 nm pitch and flat surfaces were tested) and that not only the nuclei area was affected, but also the volume and elasticity [30]. This support our findings for the L929 fibroblasts, especially for the cells cultured on the aligned fibers where the inter-fiber distances may be of similar order of magnitude. The larger nuclei size is thought to reflect a larger amount of euchromatin *i*.*e*. unpacked DNA, which should furthermore indicate a higher transcription activity of the nuclei. It could then be hypothesized that the fiber structures have induced a raised gene expression regarding cytoskeleton remodeling and possibly expression of ECM molecules as compared with the flat surfaces, although it was not tested within this study.

It has been reported that nucleus alignment on fiber substrates, due to anisotropic forces mediated from the environment, via integrins to the cytoskeleton and nucleus can have an impact not only on migration direction and speed, but also on gene and protein regulation, that eventually regulate cell behavior [31, 32]. In our study, cells on all topographies show a slight elongation of the nuclei, something that is expected at least on parallel fibers, but only on such parallel fibers do the nuclei actually align as measured. Cytoskeletal stainings show that cells cultured on the aligned fibers tend to change their orientation and elongate in the direction of the fibers, a phenomenon known as “contact guidance”, which supports our findings concerning the elliptical closure of the wound fields since the elongated shape also reflect a faster migration of cells in the direction of the long axes [33]. The fibroblasts cultured on flat and randomly oriented fiber surfaces do not show this behavior. However, the fibroblasts cultured on the more porous, randomly oriented fiber network were also found to have migrated into the 3D-fiber network, which would further explain their seemingly lower mobility in the x-y plane.

The analysis of cell mobility furthermore show that fibroblasts cultured on randomized nanofiber networks tend to spread into the wound field at a slower pace than on the other two surfaces—probably due to the multidirectional contact guidance cues from the scaffold in combination with cell infiltration into the scaffold. The wound fields on aligned fiber networks and the flat surfaces both close at a similar speed, but the wound on the aligned nanofiber surface does so slightly faster and in an elliptical formation (anisotropic/ordered cell migration), compared to the flat, and random nanofiber scaffold surfaces where the initial circular shape of the wound field is maintained (isotropic/random cell migration). These results indicate that the fibroblasts are using mechanical guidance cues provided by the fibers, to move rapidly across the wound field and thus, for regenerative purposes aligned fibers may be favorable for controlling and/or optimizing the healing process. Such migration, directed by fiber orientation, has previously been reported with other cells [34].

Finally, the results from the viability measurements show that after 48- and 96 h, cells cultured on all surfaces show good viability and similar degrees of reagent reduction. That the fibroblasts cultured on the aligned fiber networks are displaying a similar degree of reduction of the alamarBlue reagent as the fibroblasts cultured on the random nanofiber networks and flat surfaces at both evaluation points suggests that the basic survival properties of the fibroblasts are independent of scaffold fiber orientation. Note that 24 well inserts were designed without the cell stopper part, and no wounds were “induced” for these experiments. Also cell seeding concentrations were kept lower than for the mobility experiments to prevent confluence for these experiments.

In conclusion: This study shows the positive effects of contact guidance from aligned PCL nanofiber scaffolds on the healing process in a novel *in vitro* wound assay. While the topography was found to have large impact on e.g. cell polarization and migration, there was little discrepancy in cell proliferation between different topographies. We conclude that aligned fibers are beneficial for rapid wound closure and may be a suitable strategy also for *in vivo* applications.
